# Comparative Proteomic Analysis of the Hepatic Response to Heat Stress in Muscovy and Pekin Ducks: Insight into Thermal Tolerance Related to Energy Metabolism

**DOI:** 10.1371/journal.pone.0076917

**Published:** 2013-10-07

**Authors:** Tao Zeng, Xueyuan Jiang, Jinjun Li, Deqian Wang, Guoqin Li, Lizhi Lu, Genlin Wang

**Affiliations:** 1 College of Animal Science and Technology, Nanjing Agricultural University, Nanjing, People’s Republic of China; 2 Institute of Animal Husbandry and Veterinary Medicine, Zhejiang Academy of Agricultural Sciences, Hangzhou, People’s Republic of China; University of North Carolina at Chapel Hill, United States of America

## Abstract

The Pekin duck, bred from the mallard (*Anas platyrhynchos*) in china, is one of the most famous meat duck species in the world. However, it is more sensitive to heat stress than Muscovy duck, which is believed to have originated in South America. With temperature raising, mortality, laying performance, and meat quality of the Pekin duck are severely affected. This study aims to uncover the temperature-dependent proteins of two duck species using comparative proteomic approach. Duck was cultured under 39°C ± 0.5°C for 1 h, and then immediately returned to 20°C for a 3 h recovery period, the liver proteins were extracted and electrophoresed in two-dimensional mode. After analysis of gel images, 61 differentially expressed proteins were detected, 54 were clearly identified by MALDI TOF/TOF MS. Of the 54 differentially expressed protein spots identified, 7 were found in both species, whereas 47 were species specific (25 in Muscovy duck and 22 in Pekin duck). As is well known, chaperone proteins, such as heat shock protein (HSP) 70 and HSP10, were abundantly up-regulated in both species in response to heat stress. However, we also found that several proteins, such as α-enolase, and *S*-adenosylmethionine synthetase, showed different expression patterns in the 2 duck species. The enriched biological processes were grouped into 3 main categories according to gene ontology analysis: cell death and apoptosis (20.93%), amino acid metabolism (13.95%) and oxidation reduction (20.93%). The mRNA levels of several differentially expressed protein were investigated by real-time RT-PCR. To our knowledge, this study is the first to provide insights into the differential expression of proteins following heat stress in ducks and enables better understanding of possible heat stress response mechanisms in animals.

## Introduction

Recent climate warming trends are strongly affecting terrestrial biological systems, resulting in changes such as earlier timing of spring events like leaf-unfolding, bird migration and egg-laying, as well as poleward and upward shifts in habitat range for plant and animal species [1]. A number of physiological mechanisms have been reported to be important indicators of thermal stress, including synthesis of molecular chaperones, generation of reactive oxygen species (ROS), and induction of the antioxidant defense system [2–4]. To gain a comprehensive understanding of these molecular mechanisms, many transcriptomic studies have applied a systems biology approach to characterize changes in the mRNA expression of thousands of genes [5,6]. Animal adaptation to environmental stresses depends on the activation of the hypothalamic–pituitary-adrenal axis and the orthosympathetic nervous system as well as the expression of stress-related proteins. However, to our knowledge, there have been only a few studies on proteomic changes in response to heat stress in poultry, especially ducks, where aspects such as mortality, laying performance, and meat quality are severely affected.

From ancient times, domestic ducks have served as a source of food and income for people in many parts of the world, providing meat, eggs, and down feathers. We chose the Pekin duck and Muscovy duck for a comparison of proteomic changes in response to acute heat stress. The former species was bred from the mallard (*Anas platyrhynchos*) in China, and now approximately 95% of duck meat consumed in the world is that of the Pekin duck or duck species derived from Pekin duck [7]. The latter species shows a distinct genetic difference from common ducks and is believed to have originated in South America. Muscovy eggs require approximately 35 days for hatching, while only 28 days are required in the case of the Pekin duck, which is one of the reasons why the Pekin duck becomes more popular. However, Muscovy duck tolerate hot weather much better than Pekin ducks, and their offspring are sterile when crossed with common ducks. Knowledge of heat-responsive genes and proteins is therefore critical for further understanding the molecular mechanisms underlying stress tolerance. The liver, one of the most vital organs in the body, plays vital roles in the metabolism, digestion and immune defense. It has a wide range of functions to energy metabolism, such as glycogenolysis and glycogen synthesis, protein metabolism, hormone production, and detoxification. Therefore, the liver is vitally important for duck metabolism.

Proteomic analysis has become one of the best strategies for identifying the proteins and pathways that are crucial for stress responsiveness. Similar to gene expression profiling, proteomics, can analyze short-term fluctuations and help classify protein expression patterns in complicated physiological processes [8]. Recently, proteomic-based technologies have been successfully applied in many species for the functional analysis of proteomic responses to a broad range of abiotic challenges such as heat [9,10], drought [11–13], salt [14,15], cold [16], and oxidative stress [17]. However, to our knowledge, only a few studies have used MS-based proteomics to compare responses to acute and chronic thermal stress in animals [10,18].

Ducks, as waterfowl, are different from broilers in the metabolism of the liver, Until recently, only two proteomics researches have been carried out in the liver of duck, and also 73 proteins have been identified [19,20]. Some information about protein changes in the liver of duck under heat stress condition is largely unknown. In the present study, we performed comparative proteomic analysis for the liver in 2 duck species in the case of response to acute thermal stress and were able to identify the majority of the proteins that showed significant changes in expression due to changes in protein synthesis, degradation, or post-translational modification. This study offers new insights into how proteins combat the effects of high temperature and provides theoretical evidence for stress defense in animals.

## Materials and Methods

### 1. Ethics Statement

The animal care and use protocol was approved by the Institutional Animal Care and Use Committee of Nanjing Agricultural University and performed in accordance with the Regulations for the Administration of Affairs Concerning Experimental Animals (China,1988) and the Standards for the administration of experimental practices (Jiangsu, China,2008). All surgery was performed according to recommendations proposed by European Commission (1997), and all efforts were made to minimize suffering of animals.

### 2. Experimental animals and stress treatment

Pekin ducks were raised in Huzhou, Zhejiang Province, China, and the Muscovy ducks in Yuyao, Ningbo, Zhejiang Province, China. The animals were kept in a temperature-controlled room with flowing air and sufficient water and fed a standard diet 3 times daily during the experimental period. For the hyperthermia challenge, both species were maintained at 20°C for 2 weeks before processing. The temperature was increased at 10°C·h^-1^ from 20°C to 39°C ± 0.5°C. The ducks were kept at these temperatures for 1 h and then immediately returned to 20°C for a 3 h recovery period. Liver and other tissues were collected from each individual in all groups (6 Pekin ducks and 6 Muscovy ducks for all groups) and stored at −80°C until analyzed.

### 3. Protein extraction

All the chemicals used for protein separation and extraction were of analytical grade, and MilliQ water was used for all the buffers and solutions. Liver tissues were homogenized in ice-cold buffer [7 M urea, 2 M thiourea, 2% (w/v) CHAPS (cholamidopropyl-dimethylammonio-propanesulfonic acid), 50 mM DTT (dithioerithriol), 0.8% [v/v] IPG buffer pH 3–10, 1 mM PMSF (phenylmethylsulphonyl fluoride)]. The homogenates were swirled for 30 min, followed by centrifugation at 15,000*g* for 30 min at 4°C [21]. Supernatants (protein extracts) were collected, pooled, and stored at −80°C for further processing. Protein concentrations were determined with the RC DCTM kit (Bio-Rad, USA) per the manufacturer’s instructions.

### 4. Two-dimensional gel electrophoresis, gel staining, and image analysis

For this analysis, 850 µg total liver protein was loaded onto a commercially available precast IPG strip (non-linear, 17 cm, pH 3–10; Bio-Rad, USA) that was actively rehydrated at 50 V for 13 h at 20°C [22]. IEF (isoelectric focusing) was performed with voltage gradients of 250 V for 1 h, 500 V for 1 h, 2000 V for 1 h, and 8000 V for 3 h, and then run at 8000 V until a total of ~60 kVh was reached. Before performing SDS-PAGE, the focused strips were equilibrated for 15 min in 1% (w/v) DTT in equilibration buffer [50 mM Tris-HCl pH 8.8, 6 M urea, 30% (v/v) glycerol, and 2% (w/v] SDS (sodium dodecyl sulphate)] and for another 15 min in alkylating equilibration buffer that contained 1% (w/v) iodoacetamide instead of 1% (w/v) DTT. The strips were sealed on top of a 12.5% SDS-PAGE gel for electrophoresis performed using a PowerPac HV (Bio-Rad) [23].

The gel was visualized with 0.08% CBB G-250 (coomassie brilliant blue) and digitized with a high-precision scanner (VersaDoc 3000, Bio-Rad) [24]. Spot detection, measurement and matching was performed using PDQuest 2-D analysis software version 8.0 (Bio-Rad). Protein spots were matched automatically and then subjected to careful manual editing and confirmation. Spots that showed significant differences in intensity and were present on at least 2 of the 3 gels in 1 treatment were considered to be differentially expressed proteins (*p*-value≤ 0.05).

### 5. In-gel protein digestion

The gel spots selected were manually excised and destained for 30 min with 100 µL of acetonitrile (ACN) (50%) and 25 mM NH_4_HCO_3_. The gel slices were dehydrated for 10 min with ACN (100%) and completely dried in a SpeedVac. The proteins were digested in 10 µL of trypsin solution (10 ng/µL) at 4°C for 1 h and incubated at 37°C overnight. Excess trypsin was removed to prevent trypsin autodigestion. The peptides from the gel were dissolved in 30 µL of 50% (v/v) ACN [containing 2.5% (v/v) TFA (trifluoroacetic acid)]. The supernatants were collected and stored at −20°C for analysis.

### 6. Protein identification and database search

Samples were re-suspended with 5µL 0.1% TFA followed by mixing in 1:1 ratio with a matrix consisting of a saturated solution of α-cyano-4-hydroxy-trans-cinnamic acid in 50% ACN, 0.1% TFA. 1µL mixture was spotted on a stainless steel sample target plate. Peptide MS and MS/MS were performed on an ABI 4800 MALDI TOF/TOF Plus mass spectrometer (Applied Biosystems, Foster City, USA). A combined search (MS plus MS/MS) were performed using GPS Explorert^TM^ software (version 3.6, Applied Biosystems, USA). The TOF spectra were recorded in positive ion reflector mode with a mass range from 800 to 4000 Da. About eight subspectra with 60 shots per subspectrum were accumulated to generate one main TOF spectrum. Based on combined MS and MS/MS spectra, proteins were successfully identified with a 95% or higher confidence interval using the Mascot V2.3 search engine (Matrix Science, Ltd., London, UK); The other parameters were set as follows: NCBIInr-Animals database; trypsin as the digestion enzyme; one missed cleavage site; fixed modifications of Carbamidomethyl (C); partial modifications of Acetyl (Protein N-term) and Oxidation (M); 100 ppm for precursor ion tolerance and 0.3 Da for fragment ion tolerance.

### 7. Bioinformatic analysis

A BLAST homology search was performed for matches against the *Homo sapiens* protein database. The E-value was set to < 1E-5. The 10 best hits for each query sequence were taken. Of the 10 best hits, the one with the best identity matches to the query was picked as the *Homo sapiens* homolog. Gene ontology (GO) enrichment analysis and functional annotation were performed with DAVID v 6.7 (3). DAVID calculates modified Fisher’s exact p-values to determine whether a GO term is overexpressed or underexpressed in a given proteomic data set [25]. The enrichment analysis was performed separately for all 3 GO categories: biological process, molecular function, and cellular component. Approximately 90% of the *Homo sapiens* homologs were matched in the database. In addition, we performed pathway enrichment analysis by using the Kyoto Encyclopedia of Genes and Genomes (KEGG) pathway maps [26].

### 8. RNA extraction and real-time RT-PCR

Total RNA was extracted using the EasyPure^TM^ RNA Kit (TransGen Biotech Co. Ltd, Beijing, China). Its concentration and purity were determined with a spectrophotometer (NanoVue, GE Healthcare, Piscataway, NJ, USA), and the integrity was examined using 1.2% agarose gels containing 0.1% ethidium bromide. Part (1 µg) of the total RNA obtained from each extraction was reverse-transcribed in a 20 µL reaction volume by using the TransScript First-Strand cDNA Synthesis SuperMix (TransGen, Beijing, China) according to the manufacturer’s instructions. The primers were designed based on the corresponding gene sequences from *A. platyrhynchos* (Table 1). RT-PCR (relative transcriptase-PCR) was performed on an ABI 7300 instrument (Applied Biosystems, Foster City, CA, USA). The PCR conditions were as follows: 94°C for 3 min, followed by 40 cycles of denaturation at 94°C for 10 s and 60°C for 30 s. Experiments for the detection of all the genes, including β-actin, were performed in triplicate. The relative expression levels of the genes tested were calculated using the 2^−ΔΔCt^ method. Data was expressed as mean ± SEM. Statistical significance was determined using the Student’s *t* test with two-tailed *P* values. Differences were considered significant when *P* values were < 0.05.

**Table 1:** Primer sequences used for quantitative RT-PCR of the differentially expressed genes in Muscovy and Pekin ducks subjected to heat stress.

**Gene name**	**NCBI Accession number**	**Primer Sequence (5’–3’) Sense/antisense**	**Product size (bp)**
14-3-3 protein	GI55741615	CCCATAGGTATCTGGCTGAGTT TGTTGGCGGGAGTTCTGTC	115
HSP70	JQ669386	CCCCCAGATCGAGGTTACTTT CTCCCACCCGATCTCTGTTG	200
S-adenosylmethionine synthase isoform type-1	GI313760550	TGTTATTGCTGCTCTATTGG AGTCATCCTTTCCTTTGC	106
Alpha-enolase	GI213090	CTGCCTGTTCCGCTTTCA CGTTGGTTGCATCCTTCC	186
Catalase	GI71894964	CCATCCTTCATCCATAGCC GGAATACCACGGTCACTAAAC	133
HSP10	GI45384203	AGTTCCTTCCCCTGTTTGAT GCTTGTAGCACTTTCCCTTGA	109
Prohibitin	GI295148229	GTCTGTTGTGGCTCGCTTTG TCTGGCTCGTTCTGCTTCC	208
Superoxide dismutase (Cu-Zn)	GI45384217	GCAATCCTGAAGGCAAGAAG TCCACATCTGCTACTCCTCCT	121
β-actin	GI151176138	ATGTCGCCCTGGATTTCG CACAGGACTCCATACCCAAGAA	165

## Results

### 1. Identification of differentially expressed proteins

**Figure 1:**
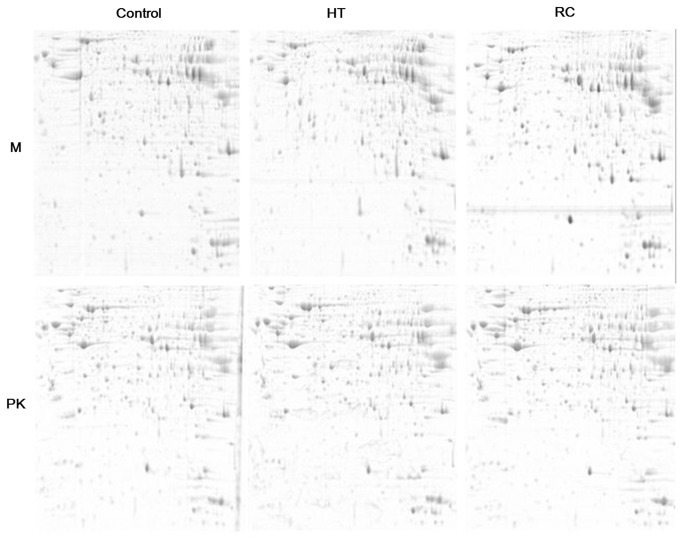
Representative spot maps of M (Muscovy) and PK (Pekin duck). **HT** stands for heat treated and **RC** stands for recovery after heat stress. Equal amounts of protein (850 µg) were loaded and separated on 17 cm IPG strips (PH 3-10), followed by electrophoresis on 12.5% SDS-PAGE gels for second dimension electrophoresis. The gels were stained with CCB G250.

**Figure 2:**
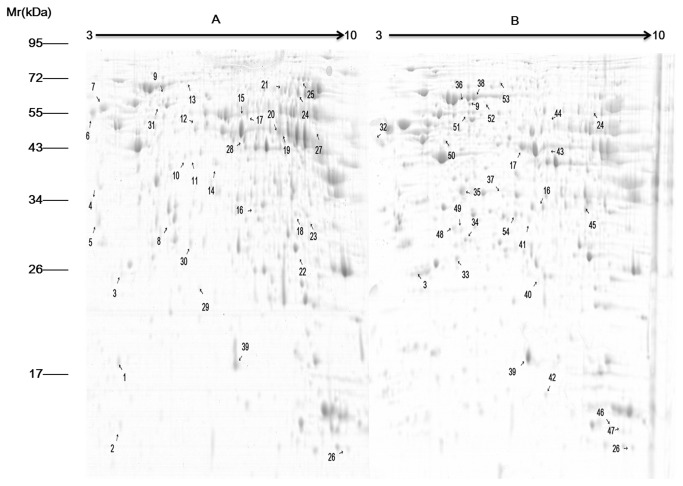
2-DE patterns of proteins extracted from Muscovy (A) and Pekin ducks (B). The experiment was repeated 3 times, and 54 differentially expressed proteins showing significant spot intensity changes under heat stress and recovery are marked in A and B. The proteins to which these 54 differentially expressed protein spots correspond are listed in Table 2.

We performed comparative visual and software-guided analysis of representative 2-DE proteome profiles of the 2 duck species before and after heat stress. The results are shown in Figure 1. In total, more than 800 protein spots were detected on at least 2 of the 3 gels for each sample. Quantitative image analysis by using the PDQuest software showed that a total of 61 protein spots indicated differential expression when compared with the control (i.e., there was more than a 2-fold difference in expression values). Spots indicating differential protein expression were excised, digested with trypsin, and analyzed by MS/MS (Figure S1). Of the 61 differentially expressed proteins, 54 were clearly identified in the NCBI database, as shown in Figure 2 and Table 2. Among these, 7 were found in both duck species, while 47 were species specific: 25 in Muscovy and 22 in Pekin.

**Table 2:** MALDI-TOF/TOF MS identification of differentially expressed proteins in Muscovy and Pekin duck livers during heat stress and recovery.

**Spot no^a^**	**Protein name**	**Species**	**Accession No^b^**		**Matched peptide sequences(m/z)^c^**	**Cov. (%)^d^**	**H. pI/Mr (kDa)^e^**	**MS^f^**	**Fold change^g^**	
									**H**	**RC**
**Cell detoxification and defense**										
1	Similar to cytochrome b5	Taeniopygia guttata	gi|224046005		FLDEHPGGEEVLR(1496.73) TLSESFIVGELHPDDR(1813.88) EQAGGDATENFEDVGHSTDAR(2204.92)	36	4.99/15.7	288	0.3±0.1	0.7±0.3(M)
24	Catalase (CAT)	Gallus gallus	gi|53127216		FPFNPFDLTK(1224.62) NFTDVHPDYGAR(1390.63) DAMLFPSFIHSQK(1519.75) IWPHGDYPLIPVGK(1590.86) GAGAFGYFEVTHDITK(1711.82) GPLLVQDVVFTDEMAHFDR(2188.06) FYTEEGNWDLVGNNTPIFFIR(2531.21)	19	8.09/60.3	568	2.2±0.4 2.1±0.3	0.8±0.1(M) 1.2±0.3(PK)
28	Peroxiredoxin 4 (PRDX4)	Taeniopygia guttata	gi|224042685		FTHLAWINTPPR(1354.71) DYGVYLEDQGHTLR(1664.78)	12	6.10/22.3	146	3.4±0.2	1.2±0.1(M)
39	Superoxide dismutase (Cu-Zn) (SOD1)	Gallus gallus	gi|45384218		LACGVIGIAK(1000.57) HVGDLGNVTAK(1109.58) GGVAEVEIEDSVISLTGPHCIIGR(2507.27)	29	6.10/16.0	217	0.5±0.0 0.5±0.0	0.9±0.0(M) 1.2±0.0(PK)
42	Peroxiredoxin 3 (PRDX3)	Meleagris gallopavo	gi|326924057		DYGVLLEGPGIALR(1471.80)	8	6.29/18.6	116	0.5±0.0	0.8±0.1(PK)
**Signal transduction**										
5	14-3-3 protein epsilon	Homo sapiens	gi|5803225		YLAEFATGNDRK(1383.68) VAGMDVELTVEER(1446.70) MDDREDLVYQAK(1523.69) AASDIAMTELPPTHPIR(1834.92)	21	4.63/29.3	456	2.0±0.3 0.5±0.0	1.1±0.2(M) 0.8±0.1(PK)
6	Calreticulin (CALR)	Gallus gallus	gi|44969651		FEPFANRDK(1138.54) KVHVIFNYK(1146.66) HEQNIDCGGGYVK(1475.65)	7	4.41/47.1	237	0.2±0.1	0.5±0.2(M)
9	Heat shock protein 70 (HSP70)	Taeniopygia guttata	gi|224083318		DAGTIAGLNVLR(1198.67) RFDDSVVQSDMK(1425.66) ARFEELNADLFR(1479.75) STAGDTHLGGEDFDNR(1690.72) TVTNAVVTVPAYFNDSQR(1980.99)	10	5.37/71.0	304	2.9±0.2 2.4±0.5	1.5±0.2(M) 0.9±0.1(PK)
26	Heat shock protein 10 (HSP10)	Gallus gallus	gi|45384204		FLPLFDR(906.50) KFLPLFDR(1034.59) VLLPEYGGTK(1075.59) VLQATVVAVGSGAR(1326.76) IVLEDKDYYLFR(1572.82)	43	8.68/11.1	250	2.4±0.5 2.1±0.2	0.9±0.3(M) 1.1±0.1(PK)
32	Calumenin	Homo sapiens	gi|2809324		EQFVEFR(953.46) WIYEDVER(1108.52) VHHEPQLSDK(1188.59) HLVYESDQNKDGK(1531.73) TFDQLTPEESKER(1578.75) DWILPSDYDHAEAEAR(1886.84) YDLFVGSQATDFGEALVR(1986.97)	26	4.47/37.1	622	2.5±0.2	1.4±0.4(PK)
34	Prohibitin (PHB)	Gallus gallus	gi|88909244		QVAQQEAER(1057.52) FDAGELITQR(1148.58) DLQNVNITLR(1184.65) ILFRPVTAQLPR(1409.85) IFTSIGEDYDER(1443.65) KLEAAEDIAYQLSR(1605.84) AAELIANSLATAGDGLIELR(1605.84) FGLGLAVAGGVVNSALYNVDAGHR(2356.23)	40	5.57/29.9	979	0.3±0.0	1.1±0.2(PK)
**Cell detoxification and defense**										
50	Rab GDP dissociation inhibitor beta-like	Meleagris gallopavo	gi|326911029	MLLYTEVTR(1124.59) GRDWNVDLIPK(1311.69) FNLPGTPPESMGR(1401.67) QLICDPSYVSDR(1451.67) MMGSEFDFEEMKR(1635.67) FLVYVANFDENDPR(1697.80) TDDYLDQPCQETINR(1866.80) SPYLYPLYGLGELPQGFAR(2140.10) NTNDANSCQIIIPQNQVNR(2198.05) NSYYGGESASITPLEDLYKR(2262.08)		30	5.44/54.5	1197	1.5±0.2	0.6±0.1(PK)
**Energy metabolism**										
2	Cytochrome c oxidase subunit Va variant 1	Taeniopygia guttata	gi|197128160	RLNFASAVR(1147.61) GINTLVGYDLVPEPK(1613.87)		17	6.74/16.2	102	0.1±0.0	0.9±0.2(M)
10	NADH dehydrogenase 1 alpha subcomplex subunit 10	Gallus gallus	gi|71895153	CPDGHSYR(990.40) GPWLEQDDVSFHHLR(1834.88)		6	6.15/41.6	163	0.4±0.0	0.5±0.1(M)
12	Alpha-enolase-like	Meleagris gallopavo	gi|326932384	IGAEVYHNLK(1142.61) YISPDQLADLYK(1424.72) VVIGMDVAASEFYR(1555.77) AAVPSGASTGIYEALELR(1803.94)		12	6.30/47.7	365	0.2±0.0	0.2±0.1(M)
13	Pyruvate carboxylase	Gallus gallus	gi|45383466	FLHECPWER(1272.57) LFLEGPTIAEEFEVELER(2120.07) VEGRPGASLPPLDFEALSQELGAR(2508.30)		4	6.26/128.0	79	2.0±0.0	1.1±0.2(M)
14	Phosphoglycerate kinase	Gallus gallus	gi|45384486	LGDVYVNDAFGTAHR(1633.78) VNEMIIGGGMAFTFLK(1726.88) ALESPERPFLAILGGAK(1767.99) VLNNMQIGNSLFDEEGSK(1993.94) QIVWNGPVGVFEWDKFSK(2135.08)		20	8.31/45.1	413	0.5±0.1	0.8±0.0(M)
17	Alpha-enolase (ENO1)	Anas platyrhynchos	gi|119338	YISPDQLADLYK(1424.72) LAQSNGWGVMVSHR(1540.76) AAVPSGASTGIYEALELR(1803.94)AGYSDKVVIGMDVAASEFYR(2177.05) SGETEDTFIADLVVGLCTGQIK(2352.15)		19	6.37/47.6	651	2.0±0.2 0.6±0.0	0.7±0.1(M) 0.3±0.1(PK)
21	Phosphoglucomutase-1	Gallus gallus	gi|84619526	IALYETPTGWK(1277.67) TGEYDFGAAFDGDGDR(1691.67) ADNFEYNDPVDGSVSR(1783.76) LSLCGEESFGTGSDHIR(1863.84) LVIGQNGILSTPAVSCIIR(2010.13)		14	8.98/67.1	460	4.2±1.4	1.0±0.3(M)
29	ATP synthase	Taeniopygia guttata	gi|224089781	AVDSLVPIGR(1025.59) ISVREPMQTGIK(1373.73) TGTAEVSSILEER(1390.69) ILGADTSAELEETGR(1560.76) TGAIVDVPVGEELLGR(1623.88) NVQAEEMVEFSSGLK(1666.79) GMSLNLEPDNVGVVVFGNDR(2131.04)		18	9.22/60.1	445	2.4±0.1	3.9±1.1(M)
31	UDP-glucose pyrophosphorylase	Homo sapiens	gi|449441	TYTFNQSR(1027.51) SFENSLGINVPR(1331.68) IQRPPEDSIQPYEK(1698.86) NENTFLDLTVQQTEHLNK(2155.09)		10	7.68/57.1	224	ap^h^	Ap
45	Aldolase B fructose-bisphosphate (ALDOB)	Anas platyrhynchos	gi|193879343	YTPQDVAAATVTTLLR(1718.92) KYTPQDVAAATVTTLLR(1847.02) ITSTTPSQLAIQENANTLAR(2128.11) GTSPLAGTNGETTIEGLDGLSER(2274.10)		31	5.80/20.3	642	0.3±0.0	0.7±0.0(PK)
**Amino acid metabolism**										
3	Catechol-O-methyltransferase isoform 1	Taeniopygia guttata	gi|224071830		LLTVEFNPEFAAIAK(1661.90)	5	5.55/30.1	113	0.2±0.0	1.0±0.1(M)
7	Prolyl-4-hydroxylase	Gallus gallus	gi|63739		LGETYRDHENIVIAK(1756.91) ILFIFIDSDHSDNQR(1818.89) SNQLPLVTEFTEQTAPK(1914.01)	9	4.66/55.2	249	0.3±0.0	0.6±0.1(M)
11	Beta-ureidopropionase	Gallus gallus	gi|50756617		NAAIANHCFTCPINR(1757.81) IPLPTDTAVAVQVAALHR(1871.06)DFELQGYGFDAAPEQLR(1954.91)AHHDLGHFYGSSYVAAPDGSR(2243.01)	18	5.76/43.3	354	0.5±0.0	1.1±0.1(M)
15	Sulfotransferase family cytosolic 1B member 1	Gallus gallus	gi|45383085		VAYGSWFDHVR(1335.64)	3	6.67/34.2	66	2.1±0.4	1.3±0.2(M)
16	S-adenosylmethionine synthase isoform type-1 (SAMS1)	Gallus gallus	gi|313760551		TACYGHFGR(1067.46) NEFSWEVPK(1134.53) TIYHLQPSGR(1170.61) NFDLRPGVIVR(1284.73) FVIGGPQGDAGVTGR(1429.73)	13	6.28/44.2	292	2.0±0.3 0.5±0.0	1.8±0.5(M) 0.8±0.0(PK)
18	Thiosulfate sulfurtransferase	Gallus gallus	gi|268370289		VLDASWYPPQER(1459.71)	4	6.80/33.1	77	2.0±0.4	1.0±0.1(M)
19	Betaine-homocysteine S-methyltransferase 1-like	Anolis carolinensis	gi|327263181		YIGGCCGFEPYHIR(1727.75) AGPWTPEATVEHPEAVR(1845.90)QGFIDLPEFPFALEPR(1874.96) AHLMSQPLAFHTPDCGK(1908.90)	15	7.18/45.6	375	2.8±0.2	0.9±0.2(M)
20	Betaine-homocysteine S-methyltransferase 1-like	Anolis carolinensis	gi|327263181		AIAEELAPER(1097.57) YIGGCCGFEPYHIR(1727.75) AGPWTPEATVEHPEAVR(1845.90)QGFIDLPEFPFALEPR(1874.96)	13	7.18/45.6	323	3.0±0.1	1.4±0.2(M)
23	Thiosulfate sulfurtransferase	Gallus gallus	gi|268370289		VLDASWYPPQER(1459.71)	4	6.80/33.1	75	1.6±0.1	1.5±0.1(M)
25	Transketolase	Gallus gallus	gi|118096822		IDSVLEGHPVPR(1317.70) LDNLVAIFDVNR(1387.75) KIDSVLEGHPVPR(1445.80) LSPALPEEDAPVVNIR(1718.92) GISGVEDKESWHGKPLPK(1963.02) LGQSDPAPLQHHVEIYQK(2059.05) TSRPENPVIYNNNEDFHIGQAK(2541.22)	15	7.27/69.2	502	2.0±0.4	1.0±0.0(M)
30	4-hydroxyphenylpyruvate dioxygenase	Gallus gallus	gi|363739843		QLHTEFSALR(1200.63) HNHQGFGAGNFK(1312.61) SLFEAIEIDQDAR(1505.74) GMQFMDVPSSYYQLLR(1949.90) FWSVDDKQLHTEFSALR(2078.02)	14	6.41/45.0	427	1.5±0.2	0.5±0.0(M)
37	Sulfotransferase 6B1	Gallus gallus	gi|118088279		FGDILFR(866.47) ELQMFPR(935.45) TGTNWLEEMVR(1334.63)	7	5.50/36.8	174	0.5±0.0	1.1±0.1(PK)
43	Adenosine kinase	Gallus gallus	gi|57529848		SGCTFPEKPDFH(1420.61) YGLKPNDQILAEEK(1616.84) FKVEYHAGGSTQNSVK(1750.86) VMPYVDVLFGNETEAATFAR(2229.08)	17	6.06/40.5	462	2.1±0.2	1.3±0.1(PK)
48	Putative methyltransferase isoform 1	Gallus gallus	gi|118093267		HVCILGHK(962.51) VVGTDISQAQIQEAK(1585.83)	8	6.00/30.6	197	1.5±0.3	0.5±0.1(PK)
54	Agmatinase	Meleagris gallopavo	gi|326932515		IYHGTPFR(989.51) GSSYAPDPYK(1083.49) CVDEGLLDCSR(1322.56)	8	5.99/36.4	229	0.6±0.0	0.2±0.1(PK)
**Proteins metabolism**										
4	Complement component 1 Q subcomponent-binding protein,	Gallus gallus	gi|363741258		QTLVLDCHYPEDEVGHEGEEESDIFTIR(3316.48)	11	4.52/27.6	48	0.1±0.0	0.9±0.1(M)
22	Hemoglobin subunit alpha-D	Cairina moschata	gi|122314		LIVQVWEK(1013.59) MFLAYPQTK(1113.55) TYFPHFDLHPGSEQVR(1928.92) SLDNLSQALSELSNLHAYNLR(2357.20)	38	7.06/15.8	271	0.2±0.0	1.3±0.2(M)
27	Protein disulfide-isomerase A3 precursor	Gallus gallus	gi|45383890		FVMQEEFSR(1187.53) EVSDFISYLKR(1355.71) GDKFVMQEEFSR(1487.67) MDATANDVPSPYEVR(1663.75)	7	5.76/56.5	175	0.4±0.0	1.3±0.4(M)
36	Cytokeratin 9	Homo sapiens	gi|435476		QGVDADINGLR(1156.58) FSSSSGYGGGSSR(1234.52) IKFEMEQNLR(1306.67) GGSGGSYGGGGSGGGYGGGSGSR(1790.72) GGGGSFGYSYGGGSGGGFSASSLGGGFGGGSR(2704.15)	14	5.19/62.3	656	2.3±0.4	1.1±0.1(PK)
38	Albumin	Anas platyrhynchos	gi|393198685		RPCFSAMGVDTK(1367.63) RHPEFSTQLILR(1495.83) AVAMITFAQYLQR(1526.79) IKDCCEKPIVER(1545.76) SFEAGHDAFMSEFVYEYSR(2270.96) QSDIETCFGEEGANLIVQSR(2252.04) IPQPDFVQPYQRPASDVICK(2357.18)	17	5.62/71.6	739	0.2±0.0	0.9±0.0(PK)
40	Proteasome subunit beta type-2 isoform 2	Gallus gallus	gi|118101652		NLADYLR(863.45) YYKPGITR(996.54) NGYELSPTAAANFTR(1610.77) APFAAHGYGAFLTLSILDR(2019.06)	24	6.07/22.8	435	2.2±0.2	1.3±0.2(PK)
41	Proteasome subunit alpha type-1-like	Meleagris gallopavo	gi|326920050		TQIPTQR(842.46) LLCNFMR(952.46) THAVLVALKR(1106.69) FVFDRPLPVSR(1331.74) IHQIEYAMEAVK(1430.72) NQYDNDVTVWSPQGR(1777.80) HMTEFTDCNLNELVK(1849.83)	28	6.46/31.3	570	1.9±0.2	1.2±0.2(PK)
44	Fibrinogen beta chain precursor	Gallus gallus	gi|267844833		QDGSVNFGR(978.45) EDGGGWWYNR(1238.51) LENAIATQTDYCR(1553.71)	6	7.84/55.2	207	2.3±0.2	3.4±0.8(PK)
46	Hemoglobin alpha A subunit	Anas platyrhynchos	gi|122347		LRVDPVNFK(1086.62) MFIAYPQTK(1097.56) IGGHAEEYGAETLER(1630.76) TYFPHFDLSHGSAQIK(1846.90)	34	8.54/15.5	375	3.2±0.3	2.4±0.2(PK)
47	Hemoglobin alpha A subunit	Anas cyanoptera cyanoptera	gi|254768512		LSDLHAQK(910.49) LRVDPVNFK(1086.62) MFIAYPQTK(1097.56) IGGHAEEYGAETLER(1630.76) TYFPHFDLSHGSAQIK(1846.90)	40	8.54/15.5	521	8.1±0.4	3.4±0.4(PK)
53	Thimet oligopeptidase-like	Meleagris gallopavo	gi|326934503		QDVYQR(807.39) GLEFDNR(849.40) INAWDMR(904.42) FYLDLYPR(1085.55) QANTGLFNLR(1132.60) KVEEMFNSR(1138.54) WDLSAEQIER(1245.60) LSEFDVEMSMR(1342.59) ALADVEVEYTVR(1363.70)	10	6.28/84.6	621	1.8±0.1	0.9±0.2(PK)
**Lipid metabolism**										
33	Apolipoprotein A-1(APO A-1)	Anas platyrhynchos	gi|546158		VVEQLSNLR(1056.59) NLAPYSDELR(1176.58) WTEELEQYR(1252.57) LREDMAPYYK(1284.62) IRPFLDQFSAK(1320.72) LRDLVDVYLETVK(1561.87) YFWQHDEPQAPLDR(1800.82)	30	5.45/28.7	495	0.3±0.0	0.6±0.2(PK)
51	Fatty acid synthase	Gallus gallus	gi|211767		QIQPEGPYR(1086.55) YSGTLHLDWVTR(1446.73) THNEYEEGLGGDYR(1638.69) GNAGQSNYGFANSAMER(1772.75) CPDLDYFVVFSSVSCGR(2006.89) TGPGEPPKLDLNNLLVNPEGPTITR(2641.41)	3	5.88/27.0	701	1.0±0.1	0.6±0.1(PK)
52	Fatty acid synthase-like	Meleagris gallopavo	gi|326930759		QIQPEGPYR(1086.55) YHGNVTLMR(1105.53) VSVHVIEGDHR(1246.64) VNAAADLITQIHK(1392.77) YSGTLHLDWVTR(1446.73) THNEYEEGLGGDYR(1638.69) GNAGQSNYGFANSAMER(1772.75)	3	5.94/27.8	533	1.0±0.3	0.4±0.0(PK)
**Cytoskeletal/structure**										
8	Capping protein muscle Z-line, beta isoform 1	Gallus gallus	gi|45382141		RLPPQQIEK(1107.64) LVEDMENKIR(1245.64) SDQQLDCALDLMR(1605.71) GCWDSIHVVEVQEK(1684.79) KLEVEANNAFDQYR(1695.82) SPWSNKYDPPLEDGAMPSAR(2217.02)	28	5.36/31.6	420	0.1±0.0	0.8±0.2(M)
35	Beta-actin	Diplodus sargus	gi|358357310		GYSFTTTAER(1131.52) QEYDESGPSIVHR(1515.70) SYELPDGQVITIGNER(1789.88) VAPEKHPVLLTEAPLNPK(1952.11)DLYANTVLSGGTTMYPGIADR(2214.06)	20	5.47/42.1	383	0.4±0.0	0.4±0.0(PK)
49	Beta-actin	Platichthys flesus	gi|7546744		IIAPPERK(922.56) GYSFTTTAER(1131.52) QEYDESGPSIVHR(1515.70) SYELPDGQVITIGNER(1789.88) DLYANTVLSGGTTMYPGIADR(2214.06)	18	5.30/42.1	437	0.5±0.2	0.2±0.0(PK)

a Spot no, spot number as given in Fig. 2.

b Accession no, accession number from the NCBI database.

c The sequences of all the identified peptides with the corresponding m/z ratio in brackets.

d Cov.: sequence coverage, number of query matched peptides.

e Isoelectric point of predicted protein/molecular mass of predicted protein.

f MS.: Mowse score, protein score was from MALDI-TOF/TOF identification. The proteins that had a statistically significant score greater than 48 (*p* < 0.05) were considered identified.

g Fold change: the ratio of the absolute intensities of protein spots in the treatment and control. H and RC represent heat stress and recovery. M and PK represent Muscovy and Pekin ducks, respectively. Each group has 6 Muscovy ducks and 6 Pekin ducks.

h ap means that this protein only appears at this stress time point.

Four main types of protein expression patterns were found during heat stress and recovery. Approximately half of the proteins belonged to a group (group I) that was significantly down-regulated under heat stress and up-regulated after recovery. This group consisted of 16 spots indicating differential protein expression from Muscovy duck and 10 from Pekin duck. These proteins might play an important role in recovery capability. The 26 group II proteins, 13 in each duck species, were up-regulated under heat stress and down-regulated after recovery. These proteins, like HSP70 and catalase (CAT), might be closely related to heat tolerance. Group III was up-regulated under both conditions; for this group, 3 spots indicating differential expression were seen for Muscovy duck and 1 for the Pekin duck. For group IV, down-regulation during both heat stress and recovery was seen, including 3 proteins for which the spots indicated differential expression only in the Pekin duck.

### 2. Comparative proteomic analyses of Muscovy and Pekin ducks under heat stress and recovery

#### 2.1. Proteins differentially expressed in both species

**Figure 3:**
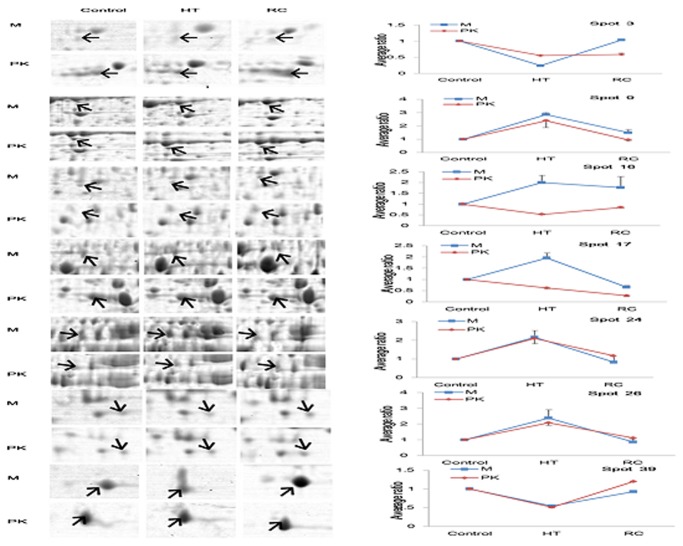
Comparison of expression patterns between Muscovy (M) and Pekin ducks (PK) for 5 important heat-responsive differentially expressed protein spots under heat stress and recovery. A: Magnified views of 5 important proteins marked in Figure 2. B: Expression patterns of the 5 proteins. Spot 9: HSP70; Spot 16: *S*-adenosylmethionine synthase isoform type-1; Spot 17: Alpha-enolase; Spot 24: catalase; Spot 39: superoxide dismutase (Cu-Zn).

Although there were similarities in the protein profiles of both species, significant differences were seen in the protein expression patterns. Of the 7 differentially expressed protein spots detected in both species, 5 (spots 3, 9, 24, 26, and 39) indicated similar expression patterns under heat stress, and 2 (spots 16 and 17) were different (Figure 3). Among the 5 with similar patterns, 2 indicated up-regulation under heat stress and down-regulation after recovery. The other 3 indicated down-regulation in both species under heat stress and up-regulation after recovery (Figure 3). Of the 2 differentially expressed protein spots with different expression patterns, *S*-adenosylmethionine synthase isoform type-1(SAMS1) (spot 16) was up-regulated under heat stress and down-regulated after recovery in Muscovy duck. In contrast, it was up-regulated under heat stress in the more heat-sensitive Pekin duck and up-regulated after recovery (Figure 3). The same expression pattern was also seen for α-enolase (ENO1) (spot 17) as well as SAMS1, but the protein level decreased in the Pekin duck after recovery.

#### 2.2. Proteins differentially expressed in only 1 species

Twenty-five and 22 heat-responsive protein spots were species specific for Muscovy and Pekin ducks (Figure 3 and Table 2). In the signal transduction group, the 14-3-3 protein (spot 5) showed obvious down-regulation under heat stress and up-regulation after recovery in Muscovy duck. The majority of proteins involved in oxidation/reduction clearly showed expression that was down-regulated under heat stress and up-regulated after recovery, including 3 in the Muscovy (spots 1, 2, and 10) and 2 (spots 42 and 51) in the Pekin duck. Two differentially expressed protein spots (spots 28 and 30) in Muscovy duck were up-regulated under heat stress and down-regulated after recovery. In the protein metabolism-related proteins, the protein disulfide-isomerase A3 precursor (spot 27) was down-regulated under heat stress and up-regulated after recovery only in Muscovy duck, whereas proteasome subunit beta type-2 isoform 2 (spot 40) and proteasome subunit alpha type-1 (spot 41) were up-regulated under heat stress and down-regulated after recovery only in the Pekin duck. Three of the differentially expressed proteins are involved in cytoskeleton organization: capping protein (actin filament) muscle Z-line, beta isoform 1 (8), beta-actin (35), and cytokeratin 9 (36). Spot 8 and spot 35 were down-regulated under heat stress in Muscovy and Pekin duck, respectively, spot 36 indicated up-regulation under stress in the Pekin duck.

### 3. Bioinformatic analysis of differentially expressed proteins

**Figure 4:**
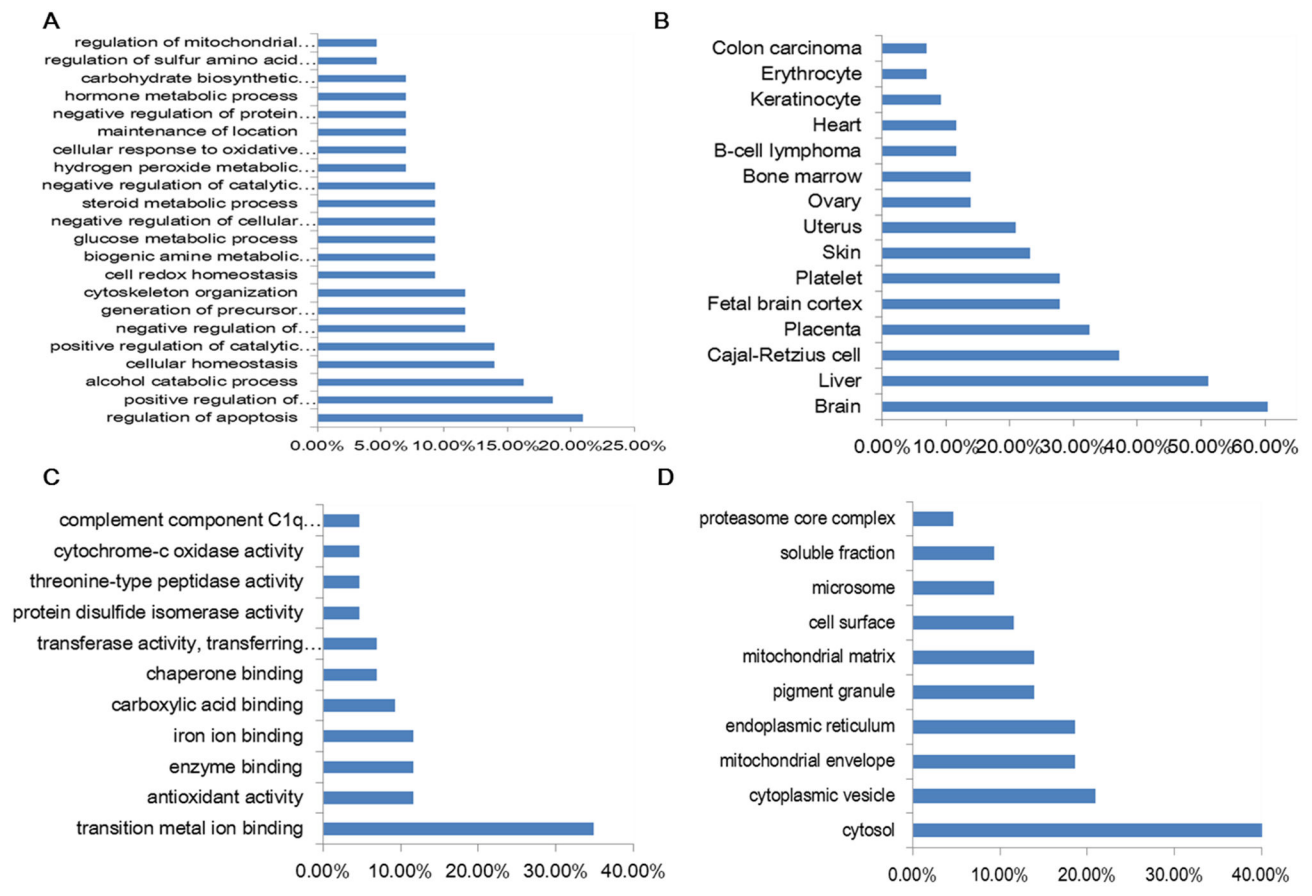
Classification of identified proteins by gene ontology (GO) enrichment analysis and tissue mapping. A: biological processes. B: tissue mapping. C: molecular function. D: cellular component.

The BLAST homology search identified 47 unique *Homo sapiens* proteins with 80.7% identity. Gene ontology analysis was performed with the 47 proteins as query, using the DAVID toolkit. It was found that 30.23% of the identified proteins are associated with positive (18.6%) and negative (11.63%) regulation of molecular function (Figure 4A). The enriched biological processes can be grouped into 3 main categories: cell death and apoptosis (20.93%), amino acid metabolism (13.95%), and oxidation/reduction (20.93%). From the 47 proteins, 42 could be matched to tissues; 50% of these were found in the liver and 60% in the brain (Figure 4B). The majority of the query proteins were associated with binding other molecules such as transition metal ions (34.88%), enzymes (11.63%), carboxylic acids (9.30%), and chaperones (6.98%) (Figure 4C). Analysis of the cellular distribution of the classified proteins showed that 42% were cytosol proteins, 20.93% were found in cytoplasmic vesicles, and 18.60% in the endoplasmic reticulum (Figure 4D).

KEGG pathway enrichment analysis of the differentially expressed proteins is the best way to perform a functional analysis. In Muscovy duck, 4 differentially expressed proteins were enriched in the 2 pathways that play a part in multiple biological processes (Table 3). Of the 4 proteins, 2 are involved in glycolysis pathway: phosphoglycerate kinase (PGK) (spot 14), ENO1 (spot 17). The other 2 are involved in antigen processing and presentation: calreticulin (CALR) (spot 6) and HSP70 (spot 9). Compared with the Muscovy duck, only one pathway which contained 2 differentially expressed proteins (ENO1 and ALDOB) was detected in Pekin duck (Table 3).

**Table 3:** Enriched KEGG pathway-based sets of differentially expressed proteins in duck liver under heat stress and recovery.

**Species**	**Pathway name**	**Count**	**Protein**
Muscovy duck	Glycolysis pathway	2	PGK (spot 14); ENO1 (spot 17)
	Antigen processing and presentation	2	CALR (spot 6); HSP70 (spot 9)
Pekin duck	Glycolysis pathway	2	ENO1 (spot 17); (ALDOB) (spot 45)

### 4. Transcription of differentially expressed proteins

**Figure 5:**
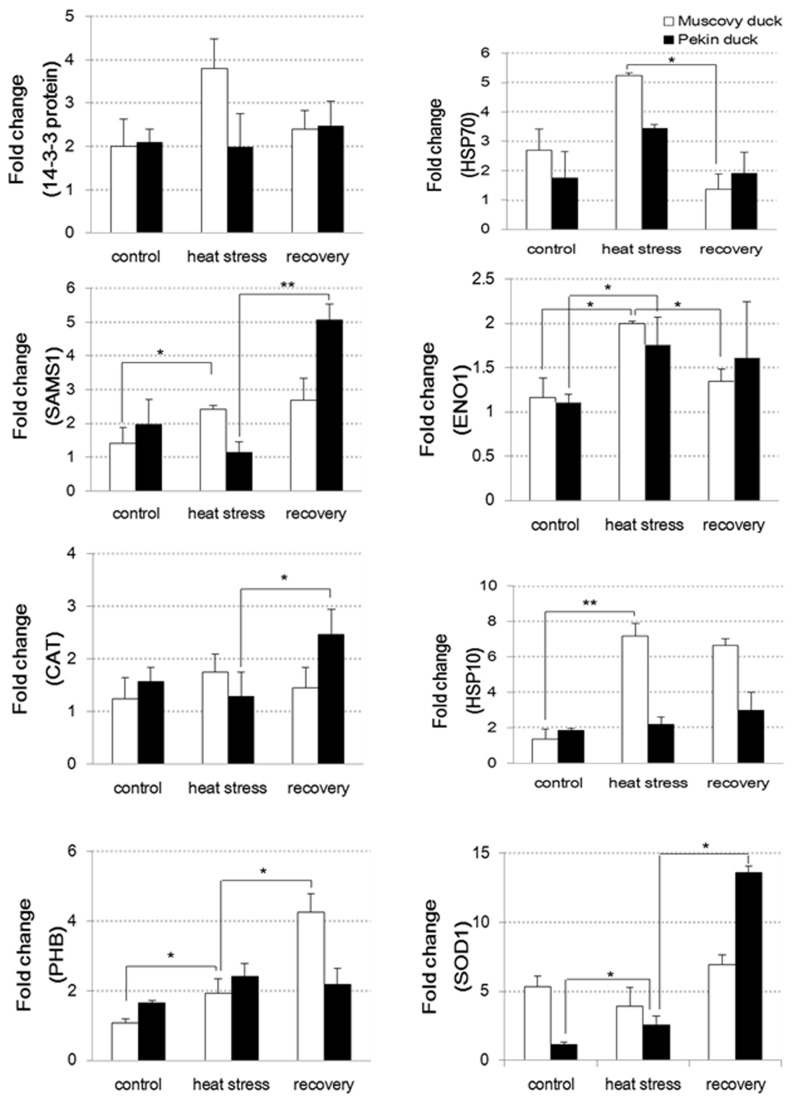
Validation of the differential expression of 8 proteins at mRNA levels by RT-PCR. The experimental procedure and the statistical estimation for the parallel runs are described in “Material and methods”. Data was expressed as mean ± SEM (6 Pekin ducks and 6 Muscovy ducks for all groups). Statistical significance was determined using the Student’s t test with two-tailed *P* values. * indicates *P* < 0.05, ** indicates *P* < 0.01. **Abbreviations: SAMS1**, *S*-adenosylmethionine synthase isoform type-1; **ENO1**, alpha-enolase; **CAT**, catalase; **PHB**, prohibitin; **SOD1**, superoxide dismutase (Cu-Zn).

To understand the relationship between changes in the levels of the identified proteins and the transcriptional levels of their encoding genes under heat stress and recovery, 8 important genes encoding the identified proteins were investigated by real-time RT-PCR. The results show that the levels of 5 of the 8 identified proteins (14-3-3 protein [spot 5], HSP70 [spot 9], SAMS1 [spot 16], CAT [spot 24], and HSP10 [spot 26]) were consistent with their mRNA expression levels (Figure 5). Gene expression levels for ENO1 (spot 17) and superoxide dismutase (SOD1) (spot 39) showed up- or down-regulation changes that correlated with their protein abundance in Muscovy duck but not in the Pekin duck. We also found that the changes in the abundance of some proteins, such as prohibitin (PHB) (spot 34), did not correspond to the mRNA expression levels of their encoding genes in both species.

## Discussion

### 1. Oxidative stress proteins

The imposition of abiotic or biotic stress can generate excess concentrations of ROS, such as superoxide (O_2_^˗^), hydrogen peroxide (H_2_O_2_), and hydroxyl radical (OH^-^) [27]. Enhanced production of ROS can disturb cellular homeostasis by causing peroxidation of lipids, oxidation of proteins, and enzyme inhibition, ultimately leading to cell death [28–30]. On the other hand, ROS also can act as signaling molecules in the regulation of processes such as cell growth, proliferation, and the stress response [31,32]. In response to the continued production of ROS, a highly efficient antioxidation system consisting of enzymic and nonenzymic elements operates to scavenge or detoxify excess ROS. This study showed that 4 proteins, that is, CAT, SOD, peroxiredoxin 3(PRDX3), and peroxiredoxin 4(PRDX4), are regulated by heat stress, which are related to ROS. CAT (spot 24) is found in almost all aerobically respiring organisms and serves to protect cells from the toxic effects of H_2_O_2_ [33]. The protein levels of CAT increased in both species, while the mRNA levels decreased in the Pekin duck. SOD (spot 39) acts as a first line of defense, converting superoxide to the less toxic H_2_O_2_. In a previous study, it was found that SOD increased after heat stress in *Mytilus galloprovincialis*, but decreased in the more heat-sensitive *Mytilus trossulus*, indicating that the 2 species differ in their cellular response to oxidative stress [10]. In this study, the protein levels of SOD decreased in both species, but the mRNA levels increased in the Muscovy duck. PRDXs (spot 28 and spot 42) can catalyze the reduction of H_2_O_2_ and alkyl hydroperoxides to water and alcohol, using reducing equivalents provided by thiol-containing proteins [34–36]. *In vitro* experiments suggested that PRDXs can become overoxidized in cases of high exposure to peroxides [37]. Another study showed that, similar to chaperones, overoxidized PRDXs may lose their catalytic function and create large molecular compounds to protect proteins from denaturation [38,39]. In our study, the PRDXs were found to be differentially expressed and showed up-regulation in Muscovy duck and down-regulation in the Pekin duck. The up-regulated PRDXs in Muscovy may contribute to superior thermotolerance by suppressing the production of active oxygen species or by preventing protein denaturation.

### 2. Signal transduction related proteins

Molecular chaperones are involved in protein folding, facilitates the normal development of the liver. HSPs have a wide range of cellular functions and are involved in processes such as protein-protein interactions, folding, and translocation; furthermore, they assist in the degradation or reactivation of damaged proteins [40,41]. In the current study, 2 differentially expressed HSPs (HSP70 and HSP10; spot 9 and spot 26, respectively) were identified, both of which were induced by heat stress. From the up-regulation of HSPs, we can infer that these proteins play a role in the defense system against heat stress. 14-3-3 protein epsilon (spot 5), one isoform of 14-3-3 proteins, a highly conserved acidic protein family that can interact with over 200 target proteins in both a phosphoserine-dependent and phosphoserine-independent manner [42], was up-regulated in Muscovy duck but down-regulated in the Pekin duck. Recent studies have made great progress toward defining the functions of 14-3-3 in cell cycle control [43], migration [44], and apoptosis [45]. Clapp et al. [46] reported that yeast cells expressing human 14-3-3β/α are able to complement a deletion of the endogenous yeast 14-3-3 and confer resistance to a variety of different stresses, including cadmium- and cycloheximide-related stress. Cell death in response to multiple stresses can be counteracted by 14-3-3 proteins. Guo et al. [15] identified three types of 14-3-3 proteins or homologues in wheat roots under salt stress. These results suggested that members of 14-3-3 protein family have diverse effects on many regulation pathways. Calreticulin (spot 6) is a major endoplasmic reticulum Ca^2+^ binding chaperone with multiple functions and is involved in a variety of cellular signaling pathways, such as those involved in innate immunity, adipocyte differentiation, apoptosis, and cellular stress responses [47,48]. Calreticulin also plays a crucial role in regulating Ca^2+^ intracellular homeostasis [49–51]. Dihaz et al. reported that calreticulin is down-regulated in the cells of the ascending limb of Henle’s loop (TALH) in response to high osmotic stress [52]. In our study, calreticulin was significantly down-regulated in Muscovy duck, but showed no change in the Pekin duck. One plausible explanation of this observation is that the decrease in calreticulin levels allows the release of free Ca^2+^ to relieve stress due to high temperatures [52]. The level of PHB decreased in the Pekin duck under heat stress and returned to normal after recovery. PHB is an evolutionarily conserved, multifunctional protein involved in cellular processes, including the regulation of proliferation, apoptosis, and transcription [53,54]. PHB (spot 34) is associated with the generation of ROS and maintaining normal respiratory function in mitochondria by reducing free radical production [55,56]. It has been shown that PHB interacts with complex Ι and subunits of cytochrome c oxidase of the respiratory chain and regulates their assembly [57]. In previous studies, it was found that PHB knockdown increased the expressions of can CAT and HSP [58], which is consistent with the regulation observed in this study, i.e., PHB was down-regulated, while CAT, HSP, PC were up-regulated.

### 3. Energy metabolism related proteins

**Figure 6:**
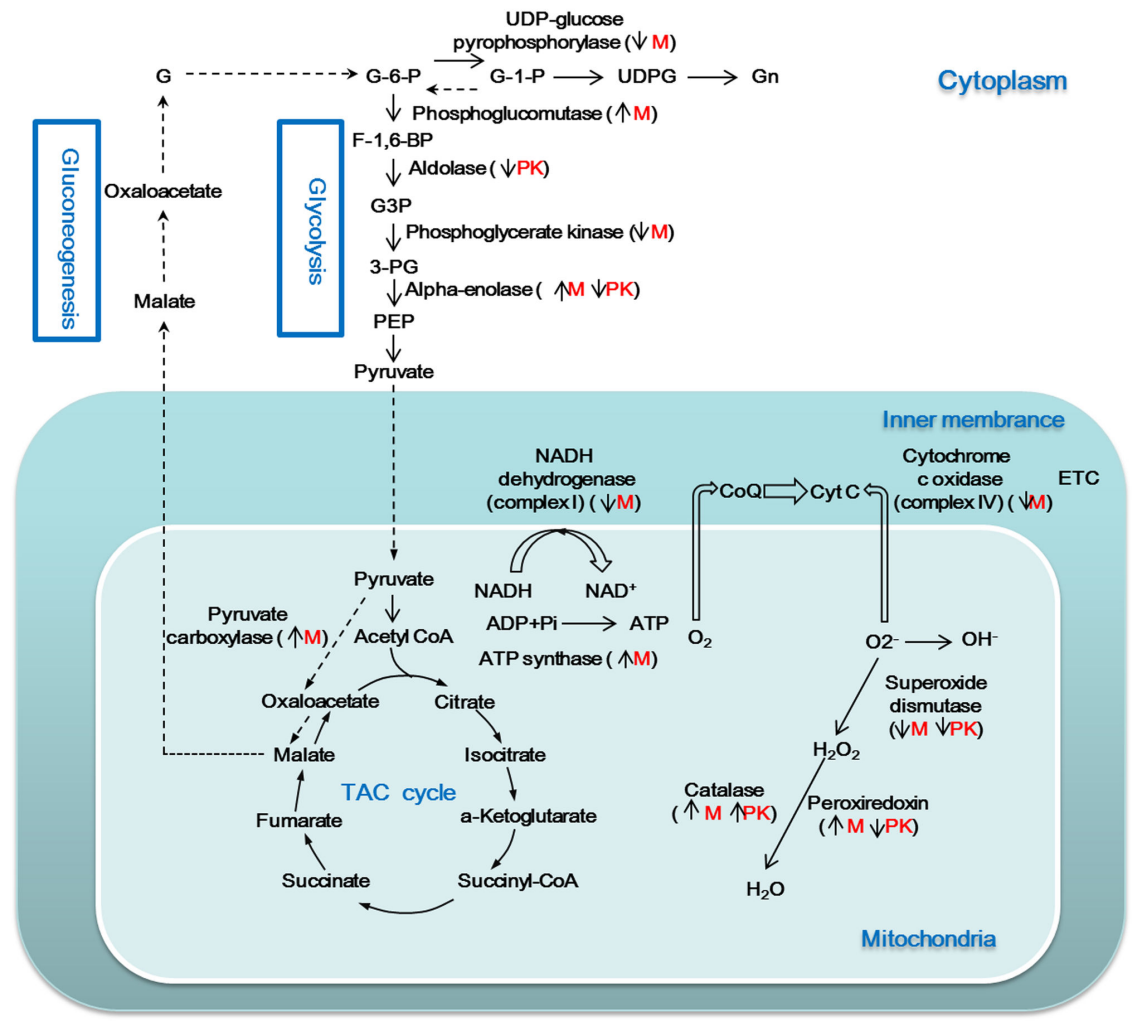
Possible energy metabolism in duck. The symbols “↑” and “↓” indicate the protein up- or down-regulation response to heat stress. Species-specific changes are indicated as M (Muscovy) or PK (Pekin duck). **Abbreviations: UDPG**, UDP-glucose; **Gn**, glycogen; **G3P**, glyceraldehyde-3-phosphate; **3-PG**, 3-phosphoglycerate; **PEP**, phosphoenolpyruvate; **CoA**, coenzyme A; **ETC**, electron transport chain.

Cytochrome *c* oxidase (spot 2) and NADH dehydrogenase (spot 10), 2 proteins involved in the electron transport chain (ETC) in mitochondria, were down-regulated by heat stress in Muscovy duck and were up-regulated after recovery. These results may be an indication of a depression in the ETC and thus ROS production. We identified spots for 2 proteins involved in glycogenolysis and glycogen synthesis in Muscovy duck; changes were observed in these spots that indicated changes in abundance in response to heat stress (Figure 6). Phosphoglucomutase-1, which transforms glucose-1-phosphate to glucose-6-phosphate in glycogenolysis, was up-regulated, while UDP-glucose pyrophosphorylase, which is involved in glycogen synthesis, was down-regulated. This regulation indicates that glycogen synthesis is suppressed and glycogenolysis is enhanced, resulting in increased glucose and, subsequently, more ATP. We feel that this indicates that Muscovy duck may have a better energy supply than the Pekin duck when subjected to heat stress. The levels of α-enolase (ENO1) (spot 17) were elevated under heat stress in Muscovy, but decreased in the Pekin duck. It has been shown that α-enolase participates in the maintenance of intracellular ATP levels in cardiomyocytes exposed to ischemic hypoxia, which is closely related to cell survival [59]. Another study found that blocking α-enolase activity with anti-enolase antibody decreases the viability of retinal cells by inducing apoptosis [60]. HSP70 (spot 9) can interact with α-enolase and protect cardiomyocytes against oxidative stress [61]. Moreover, some investigators have found that enolase 1 can regulate the differentiation and function of mouse mast cells with calreticulin (spot 6), which was also found in our study [62]. Recent proteomic studies have shown that enolase changes in response to temperature stress, but in a species-dependent manner [15,63]. These findings suggest that α-enolase is multifunctional, acting as an energy regulator and a heat stress protein. We observed an increase in α-enolase in Muscovy duck; this probably results in more energy being available to combat stress as well as to regulate apoptosis. However, this finding needs further verification.

### 4. Amino acid and protein metabolism related proteins

A total of 26 proteins related to amino acid and protein metabolism were identified; 21 up-regulated and 10 down-regulated under heat stress (Table 2). *S*-adenosylmethionine synthetase (SAMS) catalyzes the synthesis of S-adenosylmethionine (SAM) from l-methionine and ATP. SAM is an important cofactor regulating a variety of biological activities in most organisms [64,65]. In plants, SAM is used as a substrate for ethylene and polyamine synthesis in metabolic and developmental regulation [66]. In our study, the expression of SAMS1 was significantly up-regulated in the heat-stressed Muscovy duck but was down-regulated in the more heat-sensitive Pekin duck. Consistent with our results, SAMS in shoots underwent increased protein synthesis in a stress-tolerant barely cultivar and showed a decreased trend in an abiotic stress susceptible cultivar [67]. Guo et al also reported that the expression of SAM was significantly up-regulated in the salt tolerant cultivar, whereas it was down-regulated in the salt sensitive cultivar [15]. Therefore, it is most likely that an increased level of SAMS in Muscovy duck contributes to its stronger heat tolerance compare to Pekin duck. Proteasomes are large, multisubunit particles that act as the proteolytic machinery for most regulated intracellular protein breakdown in eukaryotes [68]. In our study, 2 proteasome isoforms identified in the Pekin duck showed significantly higher levels under heat stress, implying that the protein tends to be involved in protection against heat stress and oxidative damage.

The GO enrichment analysis of the differentially expressed proteins under heat stress suggests three highly enriched functional terms, i.e. cell death and apoptosis, amino acid metabolism, and oxidation/reduction. This analysis gives us a better understanding about the hepatic dynamics against thermal stress in duck. Furthermore, the consistent correlations between the protein and mRNA expression status provide us important target genes for the reverse genetic analysis through RNA interference, which may eventually contribute to the improvement of the heat resistance of ducks.

## Conclusions

To our knowledge, this study is the first to provide insights into the differential expression of proteins in liver collected from Muscovy and Pekin ducks following heat stress. The proteins identified are involved in various functions such as signal transduction, oxidative stress, and carbon, amino acid, protein and lipid metabolism. It is worth noting that some differentially expressed proteins that are regulated differently in Muscovy and Pekin ducks possibly play important roles in resisting heat stress. Expression of these proteins was also measured at the gene level. We propose that α-enolase act against heat stress and that their differential expression in Muscovy and Pekin ducks results in differences in both energy supply and apoptosis regulation. However, those conclusions were based on a small sample size, for better understanding the exact function, molecular biological analysis and other proteomic studies should be performed in the future. Further investigation will be addressed to assess the specific roles and functional correlation of these proteins as well as regulation of heat stress in ducks.

## Supporting Information

Figure S1 **Representative mass spectra of spot 17 analyzed by MALDI-TOF/TOF MS.** The differentially expressed protein spot 17 was in-gel digested by trypsin, and peptide mixture was analyzed by MALDI-TOF/TOF Proteomics Analyzer. Figure S1A) MS spectrum with tryptic peptides of spot 17, Figure S1B) MS/MS spectrum of the precursor ion with m/z 1541.85 of spot 17.
(TIF)
