# Supplementary figures and images for: Comparative Proteomic Analysis of the Hepatic Response to Heat Stress in Muscovy and Pekin Ducks: Insight into Thermal Tolerance Related to Energy Metabolism
Source: PLoS One. 2013 Oct 7;8(10):e76917. doi: 10.1371/journal.pone.0076917 (PMC3792036; doi:10.1371/journal.pone.0076917)

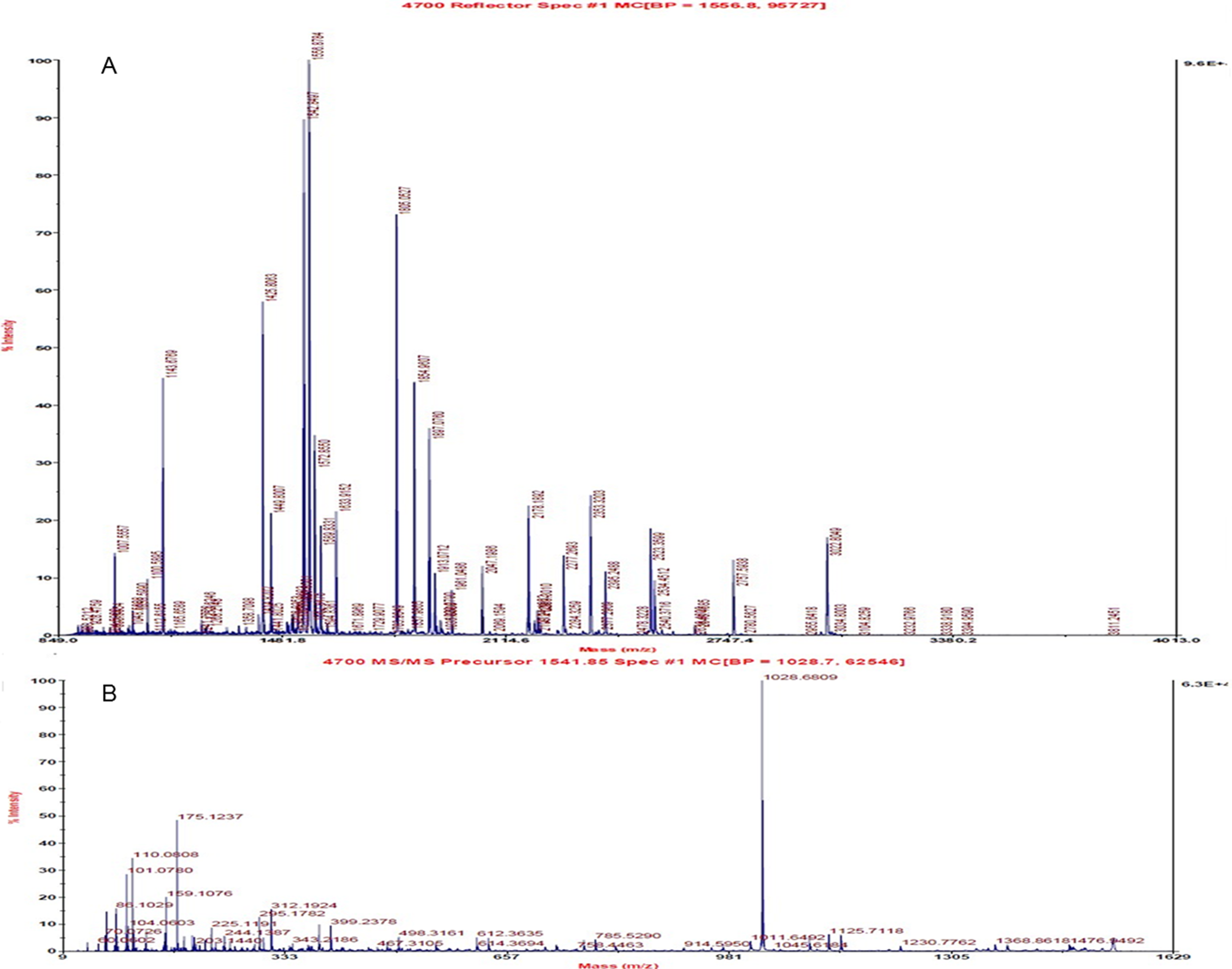

Supplement: Figure S1 — Representative mass spectra of spot 17 analyzed by MALDI-TOF/TOF MS. The differentially expressed protein spot 17 was in-gel digested by trypsin, and peptide mixture was analyzed by MALDI-TOF/TOF Proteomics Analyzer. Figure S1A) MS spectrum with tryptic peptides of spot 17, Figure S1B) MS/MS spectrum of the precursor ion with m/z 1541.85 of spot 17. (TIF) [file pone.0076917.s001.tif]
